# Successful Intra- but Not Inter-species Recombination of *msr(D)* in *Neisseria subflava*

**DOI:** 10.3389/fmicb.2022.855482

**Published:** 2022-03-30

**Authors:** Tessa de Block, Natalia González, Saïd Abdellati, Jolein Gyonne Elise Laumen, Christophe Van Dijck, Irith De Baetselier, Dorien Van den Bossche, Sheeba S. Manoharan-Basil, Chris Kenyon

**Affiliations:** ^1^Department of Clinical Sciences, Institute of Tropical Medicine, Antwerp, Belgium; ^2^Laboratory of Medical Microbiology, Vaccine and Infectious Disease Institute, University of Antwerp, Antwerp, Belgium; ^3^Department of Medicine, University of Cape Town, Cape Town, South Africa

**Keywords:** horizontal gene transfer, *msr(D)*, transformation, *Neisseria subflava*, *Neisseria gonorrhoeae*, macrolide resistance

## Abstract

Resistance acquisition *via* natural transformation is a common process in the *Neisseria* genus. Transformation has played an important role in the emergence of resistance to many antimicrobials in *Neisseria gonorrhoeae* and *Neisseria meningitidis*. In a previous study, we found that currently circulating isolates of *Neisseria subflava* had acquired an *msr(D)* gene that has been found to result in macrolide resistance in other bacteria but never found in *Neisseria* species before. To determine if this resistance mechanism is transferable among *Neisseria* species, we assessed if we could transform the *msr(D)* gene into other commensal and pathogenic *Neisseria* under low dose azithromycin pressure. Intraspecies recombination in commensal *N. subflava* was confirmed with PCR and resulted in high-level macrolide resistance. Whole-genome sequencing of these transformed strains identified the complete uptake of the *msr(D)* integration fragment. Sequence analysis showed that a large fragment of DNA (5 and 12 kb) was transferred through a single horizontal gene transfer event. Furthermore, uptake of the *msr(D)* gene had no apparent fitness cost. Interspecies transformation of *msr(D)* from *N. subflava* to *N. gonorrhoeae* was, however, not successful.

## Introduction

Transformation is one of the genetic recombination methods *Neisseria gonorrhoeae* has used to acquire resistance to every class of antimicrobials used to treat it (Unemo and Shafer, 2014). *Via* this process, *Neisseria* species are able to take up environmental DNA and incorporate it into their chromosomes (Hamilton and Dillard, 2006; Rotman and Seifert, 2014). *Neisseria* species preferably take up DNA from closely related species, especially those that use the same DNA uptake sequence (DUS) for transformation (Duffin and Seifert, 2010). An important consequence of transformation is the transfer of resistance-associated DNA fragments from commensal *Neisseria* towards pathogenic *Neisseria* (Nakayama et al., 2016; Wadsworth et al., 2018). Commensal *Neisseria* are important members of a healthy oral microbiome and hence are present in all humans (Liu et al., 2015; Tedijanto et al., 2018). This high prevalence means they are more likely to be exposed to antimicrobials used for any indication (bystander selection; Kenyon et al., 2021). As a result, commensal *Neisseria* are particularly at risk for developing antimicrobial resistance (AMR) to commonly used antimicrobials. Along these lines, recent studies have found alarmingly high minimum inhibitory concentrations (MIC) values for fluoroquinolones, macrolides and β-lactams in commensal *Neisseria* (Dong et al., 2020; Laumen et al., 2021b). Studies have confirmed that transformation of DNA from commensal *Neisseria* has played an important role in the genesis of resistance to a number of classes of antimicrobials in pathogenic *Neisseria:* macrolides *(mtrR, mtrCDE*, *rplD* and *rplY*; Wadsworth et al., 2018; Manoharan-Basil et al., 2021), β-Lactams (*penA*; Bowler et al., 1994; Ito et al., 2005), sulphonamides (*folP*) and fluoroquinolones (*gyrA*; Unemo and Shafer, 2014; Chen et al., 2020).

An additional pathway used by the pathogenic *Neisseria* to acquire AMR has been the uptake of whole genes from other species. Examples of these are the acquisition of the *tetM, ermB/C* and *bla_TEM_* genes that confer resistance to tetracyclines, macrolides and β-Lactams, respectively (Roberts et al., 1999; Unemo and Shafer, 2014). In a previous study, we identified the recent acquisition of a new ribosomal protection protein (MsrD) in *N. subflava* as a novel resistance mechanism in *Neisseria* (de Block et al., 2021). The *msr(D)* gene is part of the antibiotic resistance ATP-binding cassette F (ABC-F) protein family. The four classes of Msr proteins (A, C, D and E) operate as ribosomal protection proteins by displacing macrolides and ketolides from the ribosome. Macrolide resistance conferring *msr* genes have been identified in various species of *Streptococcus, Staphylococcus, Enterococcus, Pseudomonas* and *Corynebacterium* (Sharkey et al., 2016; Dinos, 2017). Complementation studies in these species have clearly established that *msr(D)* has a powerful effect on macrolide MICs (Daly et al., 2004; Nunez-Samudio and Chesneau, 2013; Zhang et al., 2016; Iannelli et al., 2018; Fostier et al., 2020). In our previous study, we found that the *msr(D)* in *N. subflava* was likely derived from the macrolide efflux genetic assembly (MEGA) element in *Streptococcus pneumoniae,* with whom it shared 100% sequence homology (de Block et al., 2021). As already described in other species, we found that the presence of the *msr(D)* gene in *N. subflava* was associated with higher azithromycin MICs (Iannelli et al., 2018; Fox et al., 2021).

In the current paper, we aimed to address four questions that emerged from the previous research: (1) Can the *msr(D)* gene be transformed into other strains of *N. subflava*? (2) If so, does this occur at the same insertion site? (3) Does uptake of *msr(D)* confer a fitness cost? (4) Can the *msr(D)* gene be transformed into *N. gonorrhoeae?*

## Materials and methods

### Intra- and Interspecies Transformation in Plates

The strains used in this experiment were all isolated from oropharyngeal swabs taken from men who have sex with men (MSM) attending our Sexually Transmitted Infections (STI) clinic in Antwerp, Belgium in 2019 (Laumen et al., 2021b). Nine *N. subflava* strains containing the *msr(D)* gene (azithromycin MIC ≥24 mg/L) were used as donor and two *N. subflava* and one *N. gonorrhoeae* strains without this gene were used as recipients (MIC <1 mg/L; Table 1). Genomic DNA was extracted using the EpiCentre^®^ kit. The DNA concentration (ng/μl) was determined using the NanoDrop^®^ ND-1000 spectrophotometer (Thermo Scientific). One hundred μicroliter of three different donor pools (P1–P3), each containing a mix of three donor DNA extracts of *N. subflava* (150 ng/μl), were separately mixed with 100 μl (4.0 McFarland) of the mid-log phase growth (6 h) of three recipient strains: (i) *N. subflava* (ITM_Ns_9/1: azithromycin MIC 3), (ii) *N. subflava* (ITM_Ns_45/1: azithromycin MIC 6 mg/L and (iii) *N. gonorrhoeae* (ITM_Ng_38/1: azithromycin MIC 0.19 mg/L; Table 2). Azithromycin concentration of 1.5× MIC was added as a stress factor. Control experiments did not contain azithromycin and/or DNA. The reaction mixtures were plated on blood agar and incubated for 48 h. One colony from each blood agar culture was selected for azithromycin MIC determination E-test gradient strips (bioMerieux, France). All the experiments were conducted at 36°C and 6% CO_2_.

**Table 1:** Characteristics of strains used in this study.

Isolate^*^	Species	Source of isolate	AZM MIC (mg/L)	*msr(D)*	Function in current experiment	Experiment
ITM_Ns_3/2	*N. subflava*	Laumen 2021	>256	Present	Donor Pool 1 (P1)	Transformation in Plates (Table 2) Morbidostat
ITM_Ns_27/1	*N. subflava*	Laumen 2021	24	Present	Donor Pool 1 (P1)	Transformation in Plates (Table 2) Morbidostat MIC stability
ITM_Ns_36/1	*N. subflava*	Laumen 2021	>256	Present	Donor Pool 1 (P1)	Transformation in Plates (Table 2) Morbidostat
ITM_Ns_9/2	*N. subflava*	Laumen 2021	>256	Present	Donor Pool 2 (P2)	Transformation in Plates (Table 2)
ITM_Ns_27/2	*N. subflava*	Laumen 2021	>256	Present	Donor Pool 2 (P2)	Transformation in Plates (Table 2)
ITM_Ns_29/1	*N. subflava*	Laumen 2021	>256	Present	Donor Pool 2 (P2)	Transformation in Plates (Table 2)
ITM_Ns_36/2	*N. subflava*	Laumen 2021	>256	Present	Donor Pool 3 (P3)	Transformation in Plates (Table 2)
ITM_Ns_41/1	*N. subflava*	Laumen 2021	>256	Present	Donor Pool 3 (P3)	Transformation in Plates (Table 2) Template for PCR transformation
ITM_Ns_49/1	*N. subflava*	Laumen 2021	>256	Present	Donor Pool 3 (P3)	Transformation in Plates (Table 2)
ITM_Ns_9/1	*N. subflava*	Laumen 2021	3	Absent	Recipient	Transformation in Plates (Table 2) MIC stability Growth curve
ITM_Ns_45/1	*N. subflava*	Laumen 2021	6	Absent	Recipient	Transformation in Plates (Table 2)
ITM_Ng_38/1	*N. gonorrhoeae*	Laumen 2021	0.19	Absent	Recipient	Transformation in Plates
ITM_Ng_21.021	*N. gonorrhoeae*	Clinical sample	1	Absent	Recipient	Morbidostat
WHO-X	*N. gonorrhoeae*	Reference strain	0.004	Absent	Recipient	PCR transformation

* *Ns: N. subflava; Ng: N. gonorrhoeae.*

**Table 2:** MIC values after intraspecies (*N. subflava*) transformation in plates.

		Intraspecies recombination in *N. subflava*					
		Recipient 1 (ITM_Ns_9/1)			Recipient 2 (ITM_Ns_45/1)		
		Sample	MIC AZM^1^	*msr(D)* qPCR	Sample	MIC AZM^1^	*msr(D)* qPCR
Transformation experiments	Donor DNA *N. subflava* P1	ITM_Ns_9/1_P1^2^	>256	Pos^3^	ITM_Ns_45/1_P1^2^	>256	Pos
	Donor DNA *N. subflava* P2	ITM_Ns_9/1_P2	>256	Pos	ITM_Ns_45/1_P2	>256	Pos
	Donor DNA *N. subflava* P3	ITM_Ns_9/1_P3	>256	Pos	ITM_Ns_45/1_P3	>256	Pos
Control experiments	AB control	ITM_Ns_9/1_AZM	4	Neg	ITM_Ns_45/1_AZM	1	Neg
	DNA control	ITM_Ns_9/1_DNA	1.5	Neg	ITM_Ns_45/1_DNA	3	Neg
	Growth control	ITM_Ns_9/1	1.5	Neg	ITM_Ns_45/1	2	Neg

1 *Post-transformation minimum inhibitory concentrations of azithromycin (MIC AZM) in mg/L*.

2 *Transformed stains subjected to whole-genome sequencing*.

3 *Pos: positive confirmation of msrD transformation by qPCR and Neg: negative confirmation*.

### Inter-species Transformation in Morbidostat

The transformation experiment was performed in a NGmorbidostat. The construction, optimalisation and use of the NGmorbidostat have been described in detail elsewhere (Verhoeven et al., 2019; Laumen et al., 2021a). In brief, the NGmorbidostat is a bioreactor that measures bacterial growth *via* optical density measurements and is used to assess the evolution of antimicrobial resistance (AMR) over time within a constant temperature (35°C–36°C) and CO_2_ range (5.5%–6%). In this experiment, we only used the incubator and turbidity measurement functions with the programme MATLAB, to record the growth rate of *N. gonorrhoeae* (The Math Works, Inc. MATLAB, version R2015b).

The experiment was conducted in four flasks with a total volume of 15 ml in each of gonococcal (GC) broth supplemented with (1%) IsoVitaleX, henceforth referred as GC medium. The conditions were as: (1) 1.5× MIC azithromycin + DNA from *msr(D)* containing *N. subflava*, (2) 1.5× MIC azithromycin, (3) DNA from *msr(D)* containing *N. subflava* and (4) GC medium (Supplementary Figure 1). To achieve this, firstly we added 200 μl of *N. gonorrhoeae* (ITM_Ng_21.021 with Azithromycin MIC 1 mg/L; Table 1) at 4.0–5.0 McFarland in all four flasks. After 6 h, the growth curve reached the mid-log phase and 100 μl of HLR-Azithromycin DNA from pool 1 of *N. subflava* (150 ng/μl) was added to flasks 1 and 3. At the same time point, azithromycin was added to a final concentration of 1.5 mg/L in flasks 1 and 2. After 24 h, 7.5 ml of the old medium was replaced by fresh medium and an additional 100 μl of HLR-Azithromycin DNA from *N. subflava* (150 ng/μl; flasks 1 and 3) and 1.5 mg/L of azithromycin of the ITM_Ng_21.021 was added (flasks 1 and 2; Table 1). This process was repeated daily for 7 days, after which the azithromycin concentration was increased to 3 mg/L for another 7 days.

### Inter-species Transformation With *msr(D)-*DUS DNA Fragment

*Msr(D)* was PCR amplified from *N. subflava* isolate ITM_Ns_41/1 (Table 1) using primers containing a AT-DUS tag to facilitate inter-species transformation to *N. gonorrhoeae*, forward primer (5’-GAT GCC GTC TGA ACA AAT GAT AAC TGA GG-3’) and reverse primer (5’-GAA TCA ATA CTG ACC AGC GAC-3’). This amplification was carried out as a touchdown PCR: the initial denaturation consisted of 5 min at 95°C, followed by amplification for 10 cycles at 94°C for 30 s, 55°C for 30 s and 72°C for 3 min. The next stage consisted of 35 cycles, lasting 5 more seconds at each cycle, at 94°C for 30 s, 60°C for 30 s and 72°C for 3 min. A final extension step was carried at 72°C for 7 min. The PCR fragment size was analysed on an agarose gel. The concentration of the amplicon was determined using the NanoDrop^®^ ND-1000 spectrophotometer (Thermo Scientific). The PCR product was used for transformation using the ‘*Transformation in plates*’ methodology as described above with 100 μl (150 ng/μl) as DNA donor.

### Confirmation of *msr(D)* Transformation With qPCR

Presence or absence of the *msr(D)* gene in transformant strains were confirmed using quantitative PCR (qPCR). The DNA of the recipient strains was extracted using the EpiCentre^®^ kit. The primers used to amplify the internal region of the *msr(D)* (637–934) were as: Forward (5’-GCG GAG GAA AAG CGA AAA C-3’) and Reverse (5’-ACA GAG CCT TAT CCC CAA ATAC-3’). The master mix was composed by 10× EHF PCR buffer (5 μl), 2 Mm dNTPs (7 μl), 5 μM Primer Forward (3 μl), 5 μM Primer Reverse (3 μl), 3.5 U/μl EHF Taq Polymerase (0.5 μl), Rnase free water (21.5 μl) and DNA (10 μl).The qPCR protocol consisted of an initial denaturation stage at 95°C during 5 min followed by amplification for 45 cycles of 94°C for 30 s, 55°C for 30 s and 72°C for 3 min. This step was followed by the final stage consisting of a single cycle of 72°C for 7 min. The specificity of the amplicon was confirmed by conducting melting point analyses.

### Assessment of Fitness Cost Based on MIC Stability

To test the stability of the transformed *N. subflava*, a single colony of HLR-azithromycin *N. subflava* strain (ITM_Ns_27/1; azithromycin MIC of 24 μg/ml) and a single colony of one transformant strain of *N. subflava* (ITM_Ns_9/1; azithromycin MIC of 256 μg/ml) were subcultured every 24 h in blood agar plates without additional azithromycin for 7 days, similar to the one described in O’Regan et al. (2010). The azithromycin MICs were tested daily on a single colony from each plate with E-tests (Table 3).

**Table 3:** Observation of the azithromycin MIC evolution in ITM_Ns_27/1 donor and ITM_Ns_9/1_P1 transformant strain (both *Neisseria subflava*) after serial subculturing in plates with absence of azithromycin as stress factor.

Isolate	Day 1 (AZM MIC) (mg/L)	Day 2 (AZM MIC) (mg/L)	Day 3 (AZM MIC) (mg/L)	Day 4 (AZM MIC) (mg/L)	Day 5 (AZM MIC) (mg/L)	Day 6 (AZM MIC) (mg/L)	Day 7 (AZM MIC) (mg/L)
ITM_Ns_27/1	24	16	16	32	16	12	16
ITM_Ns_9/1	>256	>256	>256	>256	>256	>256	>256

### Evaluation of Fitness Cost in Transformants by Growth Curves Rate Variance

The NGmorbidostat was used to compare the growth curves of *N. subflava* recipient and transformant strain. In a total volume of 15 ml of GC broth supplemented with 1% IsoVitalex (BD BBL™) for each experiment, 100 μl of a 4.0 McFarland suspension in PBS of *N. subflava* recipient (ITM_Ns_9/1) or *N. subflava* transformant (ITM_Ns_9/1transformant) strain was added in triplicate. The growth curves were assessed for 18 h, *via* measurement of optical density every 20 min. Difference in growth curves was assessed *via* analysis with R (R Core Team, 2019) package ‘growthcurver’ (Sprouffske and Wagner, 2016) with the data obtained from the NGmorbidostat. R was also used to perform the t-test on the samples to confirm or deny or null hypothesis and to obtain the value of *p* (Supplementary Figure 2).

### Whole-Genome Sequencing

For whole-genome sequencing (WGS) analysis, the following samples were chosen: (i) DNA recipients after transformation (ITM_Ns_9/1, ITM_Ns_45/1) and (ii) Transformation in morbidostat ITM_Ng_21.021 (Time points 1, 7 and 14 for AZM + DNA and day 14 for the controls). Genomic DNA was extracted using the MasterPure Complete DNA and RNA Purification Kit (Epicentre, Madison, Wisconsin, United States) and suspended in nuclease-free water. Indexed paired-end libraries were prepared using the Nextera XT DNA Library Prep Kit (Illumina, San Diego, CA, United States) and sequenced on an Illumina MiSeq instrument (Illumina, San Diego, CA, United States). Data are available in GenBank: https://www.ncbi.nlm.nih.gov/sra/PRJNA794044. Processed Illumina reads were *de novo* assembled with Shovill (v1.0.4; https://github.com/tseemann/shovill) which uses SPAdes (v3.14.0) using the following parameters: --trim --depth 150 --opts --isolate (Prjibelski et al., 2020). The quality of the contigs was verified with Quast (v5.0.2; Gurevich et al., 2013) followed by annotation using Prokka (v1.14.6; Seemann, 2014). WGS assemblies of the donor (ITM_Ns_3/2, ITM_Ns_27/1 and ITM_Ns_36/1) and recipient strains (ITM_Ns_9/1, ITM_Ns_45/1 and ITM_Ns_38/1) were available from a previous study by our group and included in the comparative analysis (de Block et al., 2021). BLAST Ring Image Generator (BRIG) was used for genome comparison (Alikhan et al., 2011). Mauve (Darling et al., 2004) was used to align contigs and MEGAX (Kumar et al., 2018) was used to align DNA fragments. Percent sequence identity of DNA fragments was calculated using Muscle (https://www.ebi.ac.uk/Tools/msa/muscle/, version 3.8.31).

## Results

### Horizontal Gene Transfer of *msr(D)* From Commensal *Neisseria*

#### Intra-species Transformation of *msr(D)* on Agar Plates

After 48 h of exposure to each of the three pools of high-level resistance (HLR)-azithromycin DNA (donor) on agar plates, both *N. subflava* recipient strains (ITM_Ns_9/1 and ITM_Ns_45/1; Table 1) attained an azithromycin MIC >256 mg/L (*n* = 6; Table 2). These isolates are henceforth referred to as transformants. There was no increase in azithromycin MIC in the control experiments. To confirm if the uptake of the *msr(D)* was successful in these six transformants, the presence of *msr(D)* was confirmed using qPCR (Table 2). One transformant strain of each recipient was used for WGS.

#### Inter-species Transformation of *msr(D)* on Agar Plates

In the three experiments where *N. gonorrhoeae* was used as recipient, the azithromycin MIC did not increase following incubation with the three donor DNA pools. qPCRs confirmed that *msr(D)* was not taken up by *N. gonorrhoeae* in any of these experiments (ct value >30 or NA).

### Transformation of *Neisseria gonorrhoeae* in the NGmorbidostat

**Figure 1:**
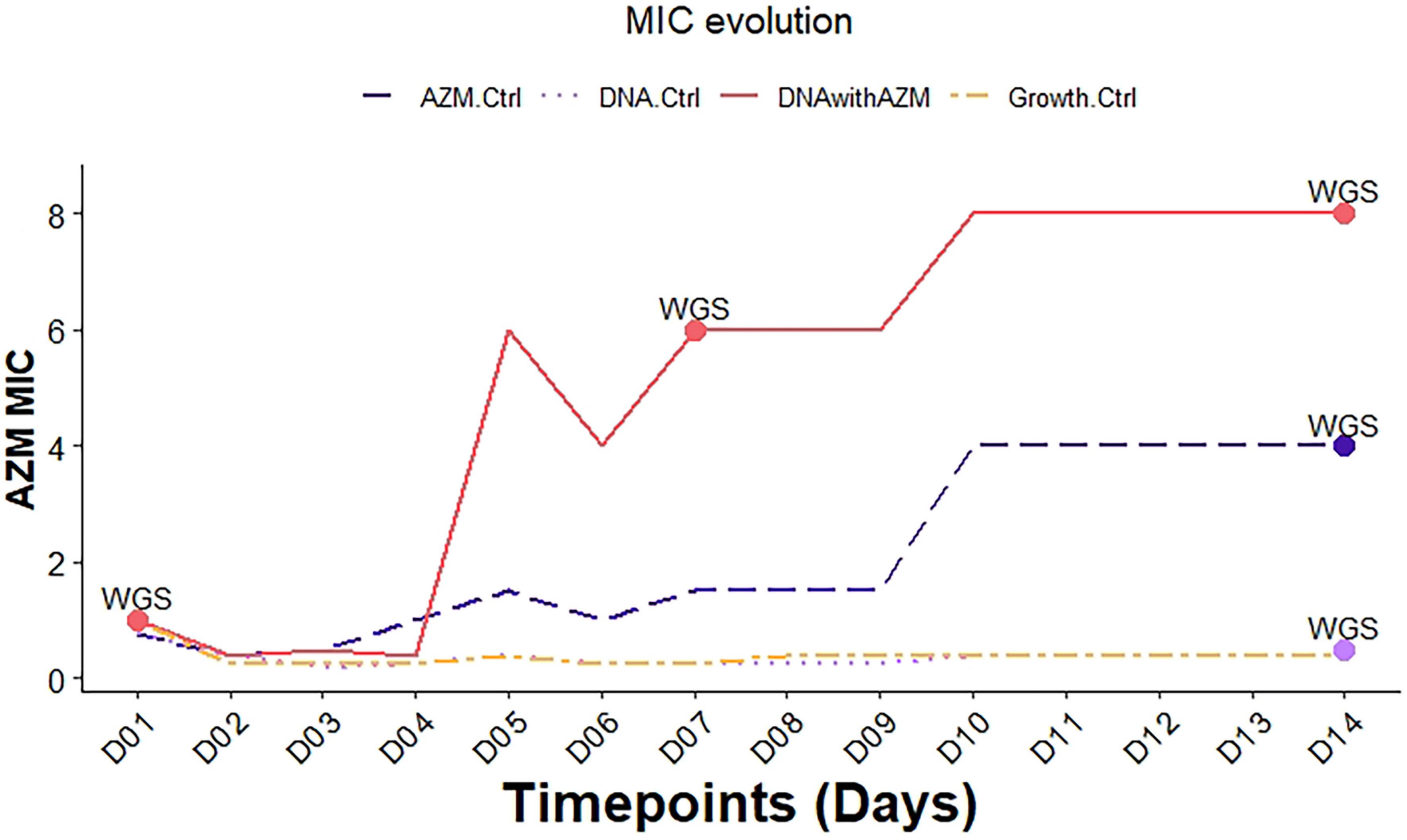
Azithromycin MIC evolution of *Neisseria gonorrhoeae* in the morbidostat transformation experiment in different conditions. (AZM.Ctrl: azithromycin control (condition 2); DNA.Ctrl: DNA control (condition 3); DNAwithAZM: DNA with azithromycin (condition 1); Growth.Ctrl: Growth control (condition 4); WGS—whole-genome sequencing, D01—day 1, etc., MIC—minimal inhibitory concentrations). The time points when samples were subjected to WGS are indicated with a dot.

Differences were noted in the azithromycin MIC trajectories in the four flasks (Figure 1). The azithromycin MIC of the *N. gonorrhoeae* recipient increased by day 5 in the flask containing DNA + azithromycin (condition 1) However, the qPCR of *msr(D)* remained negative in all samples. WGS of samples from day 7 and day 14 revealed a well-known mutation previously linked to macrolide resistance in *N. gonorrhoeae*: C2611T (*Escherichia* coli numbering) in the 23S rRNA gene.

WGS of the *N. gonorrhoeae* recipient in the azithromycin control on day 14 (condition 2) revealed that the recipient acquired the recently described macrolide resistance-associated mutation (RAM) G70D in the 50S ribosomal protein L4 (*rplD*; Ma et al., 2020; Laumen et al., 2021a).

There was no increase in azithromycin MICs of the *N. gonorrhoeae* recipient in the DNA control (condition 3) and the growth control (condition 4).

### Transformation of *Neisseria gonorrhoeae* With *msr(D)* PCR Product

There was no increase in the azithromycin MIC of the *N. gonorrhoeae* recipient strain after incubation on agar plates for 48 h with a dsDNA fragment containing *msr(D)* and a DUS. qPCR analysis confirmed that the *msr(D)* gene was not acquired by the recipient (ct value >30).

### Fitness Cost of Transformants

#### MIC Stability

There was no significant decline in the azithromycin MIC value in the transformant or donor strain during 7 days of subculturing (Table 3).

#### Growth Rate

There was no statistically significant difference obtained in the growth rate ratios between recipient (ITM_Ns_9/1, mean: 0.64) and transformant strain (ITM_Ns_9/1_P1, mean: 0.67; value of *p*: 0.3673; Supplementary Figure 2).

### Whole-Genome Sequencing of *msr(D)* Transformants

**Figure 2:**
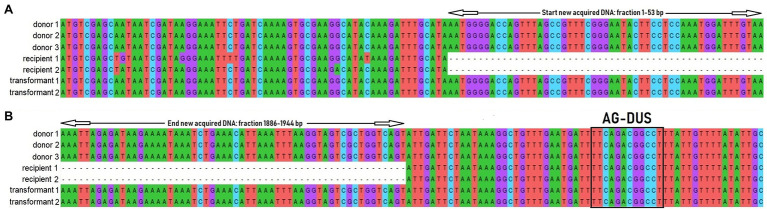
Fragment of DNA sequence alignment of the start **(A)** and end **(B)** point (black triangle) of the integration of the new acquired DNA fragment containing *msr(D)*. AG-DUS 31 bp upstream *msr(D)* is indicated with a black box. Transformation of recipient strains (recipient 1; ITM_Ns_9/1, recipient 2; ITM_Ns_9/1) with donor DNA containing *msr(D)* (donor 1; ITM_Ns_3/2, donor 2; ITM_Ns_27/1 and donor 3; ITM_Ns_36/1) resulted in transformant 1 (ITM_Ns_9/1_P1) and 2 (ITM_Ns_45/1_P1).

**Figure 3:**
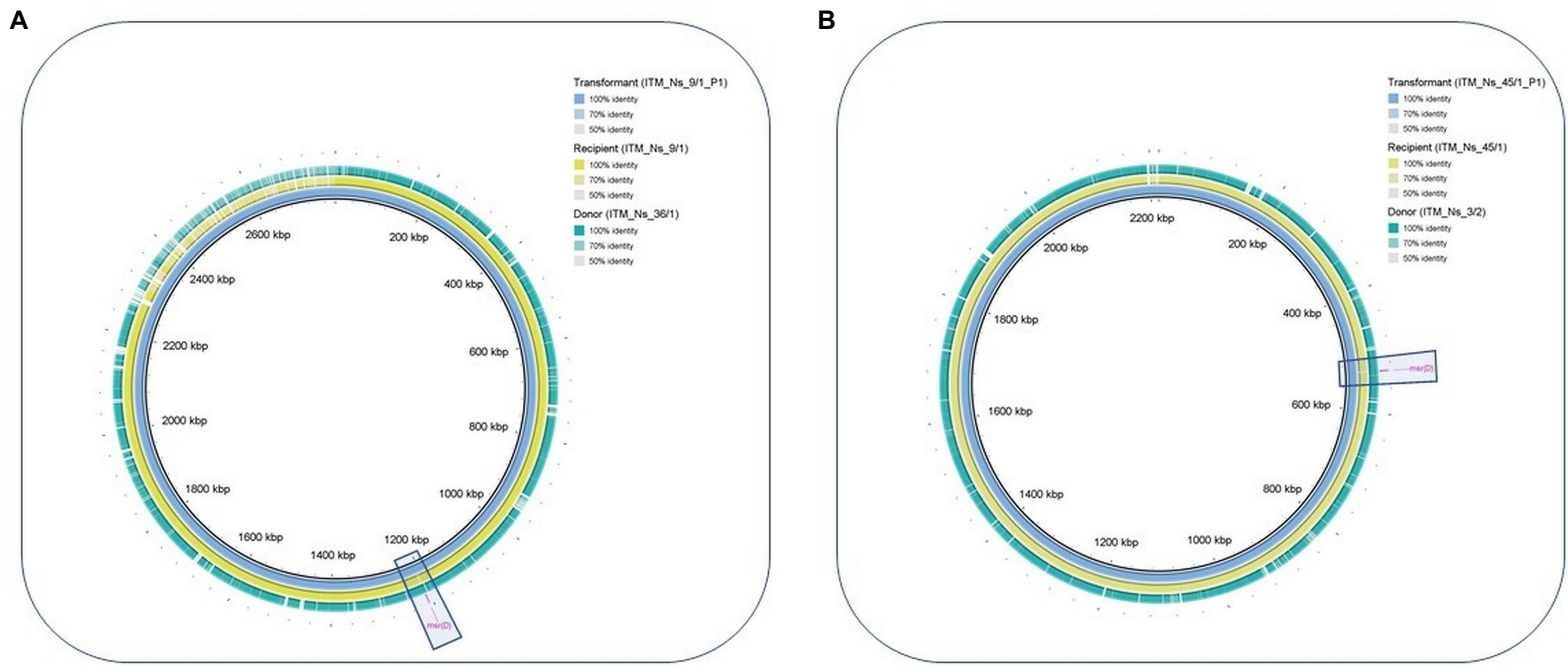
Genome visualisation of *msr(D)* transformation between two strains of *N. subflava* in Brig (**A**; transformant ITM_Ns_9/1_p1 used as reference with recipient ITM_Ns_9/1 and donor ITM_Ns_36/1, **B**; transformant ITM_Ns_45/1_P1 used as reference with recipient ITM_Ns_45/1 and donor ITM_Ns_3/2). The inner-ring (blue) depicts the transformant genome, where *msr(D)* (manually labelled in fuchsia) is integrated in the recipient (yellow circle) from donor (green circle).

Two PCR-confirmed *msr(D)*-transformant *N. subflava* strains (ITM_Ns_45/1_P1 and ITM_Ns_9/1_P1; Table 2) were subjected to WGS to identify the exact integration site of *msr(D).* DNA sequences including the upstream (6,725 bp) and downstream (9,134 bp) region of *msr(D)* with a maximum total length of 17,803 bp were extracted for further analyses. Donor (ITM_Ns_3/2, ITM_Ns_27/1 and ITM_Ns_36/1), recipient (ITM_Ns_45/1 and ITM_Ns_9/1) and transformant (ITM_Ns_9/1_P1 and ITM_Ns_45/1_P1) DNA sequences were aligned. This alignment revealed the acquisition of the *msr(D)* gene at the same site (GCATA-acquisition of *msr(D)-*ATTGA) in the chromosome in both recipients, 32 bp downstream of a DUS sequence (Figure 2). Genome comparison of donor, recipient and transformant revealed that the transformants had acquired a new *msr(D)-*containing DNA fragment, which originated from the donor, and was not present in the recipient (Figure 3).

**Figure 4:**
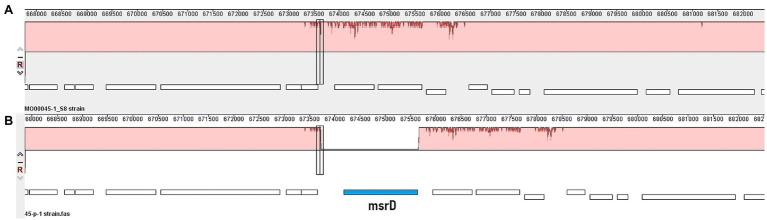
Mauve alignment of recipient (**A**; ITM_Ns_45/1) with transformant (**B**; ITM_Ns_45/1_P1) strain. Red color indicates similar DNA sequence in both strains, the blue box indicates the *msr(D)* gene and the vertical bar indicates the integration site in the recipient strain where the DNA fragment containing *msr(D)* was inserted. The rough lane in the red box indicates differences in DNA sequence between the two isolates and thus depicts the complete fragment size in the transformant strain (ITM_Ns_45/1_P1) which originated from the donor strain.

A more global alignment conducted in Mauve illustrates the chromosomal organisation around the acquired *msr(D)* in the transformant ITM_Ns_45/1_P1 compared to the recipient strain (Figure 4). SNP analysis revealed that transformant ITM_Ns_9/1_P1 had taken up a larger DNA fragment than transformant ITM_Ns_45/1_P1 ((Figure 2; Table 4; Supplementary Table 1). The length between the first and last SNP of transformants compared to the recipient strains was 12,033 bp and 5,113 bp for transformants ITM_Ns_9/1_P1 and ITM_Ns_45/1_P1, respectively. The acquired DNA extended from upstream of *msr(D)* (7,234 bp in ITM_Ns_9/1_P1; 2,883 bp in ITM_Ns_45/1_P1) to downstream of *msr(D)* (3,335 bp in ITM_Ns_9/1_P1, 766 bp in ITM_Ns_45/1_P1; Supplementary Table 1).

**Table 4:** Characteristics of integrated DNA fraction in transformant ITM_Ns_9/1_P1 and ITM_Ns_45/1_P1.

Transformant strain	Integrated DNA fraction			% Identical to donor strain		
	Upstream *msr(D)*	Downstream *msr(D)*	Complete length	ITM_Ns_3/2	ITM_Ns_27/1	ITM_Ns_36/1
ITM_Ns_9/1_P1	7,234 bp	3,335 bp	12,033 bp	97.57	92.78	99.96
ITM_Ns_45/1_P1	2,883 bp	766 bp	5,113 bp	99.92	92.41	98.43

The uptake-fragment of transformant ITM_Ns_9/1_P1 showed high similarity to ITM_Ns_36/1 (97.57% identical to ITM_Ns_3/2, 92.78% to ITM_Ns_27/1 and 99.96% to ITM_Ns_36/1) and the uptake-fragment of transformant ITM_Ns_45/1_P1 showed high similarity to ITM_Ns_3/2 (99.92% identical to ITM_Ns_3/2, 92.41% to donor ITM_Ns_27/1 and 98.43% to donor ITM_Ns_36/1; Table 4). These data suggest that for both transformants, the complete *msr(D)* containing fragment was taken up from a single (but different) donor in a single transformation event (ITM_Ns_3/2 as the donor for ITM_Ns_45/1 and ITM_Ns_36/1 for ITM_Ns_9/1_P1).

## Discussion

We studied the intra- and inter-species transformability of the resistance conferring *msr(D)* gene in *Neisseria* spp. We found that intraspecies transformation in commensals under azithromycin pressure in *N. subflava* was very efficient. Azithromycin triggered the integration of *msr(D)* into strains of *N. subflava* with low level azithromycin resistance (3–6 mg/l). The *msr(D)* gene could be acquired without any apparent fitness cost and was universally associated with an elevation of azithromycin MICs to >256 mg/L. We did not conclusively establish that *msr(D)* is responsible for macrolide resistance in *N. subflava*. This was, however, not one of the study aims as this has been clearly established for a range of gram negative and positive bacterial species (Daly et al., 2004; Nunez-Samudio and Chesneau, 2013; Zhang et al., 2016; Iannelli et al., 2018; Fostier et al., 2020).

In a previous study, we found that nine out of 11 clinical *N. subflava* strains had the *msr(D)* gene integrated in the same place in the genome (de Block et al., 2021). The complete integrated DNA sequence originates from the MEGA element in *S. pneumoniae*. The integration in *N. subflava* was located 32 bp downstream of a DUS sequence, suggesting that this DUS enhances the transformation efficiency. WGS of transformant *N. subflava* strains in the current study revealed that the chromosomal integration of the *msr(D)* gene was integrated into the same position in the genome as the donor strains. The complete fragment size in the recipients included up- and downstream regions of *msr(D)* with a total length of 5 and 12 kb, respectively. Thus, a DNA insert up to 12 kb can be transformed into the cell and integrated into the chromosome in a single event. Other studies have found similar sized transformation events in *Neisseria* spp. (Chen et al., 2020). A previous core genome MLST analysis revealed that the *msr(D)* gene was present in different clusters of clinical isolates of *N. subflava* (de Block et al., 2021). This implies that horizontal gene transfers such as transformation either took place on more than one occasion, or that the *msr(D)* has been taken up and lost in sub-lineages. This suggests that single transformation events of *msr(D)* could also take place *in vivo*.

The MEGA element in *S. pneumoniae* contains both the *msr(D)* gene (which is responsible for displacing bound macrolides) and *mef(A)* which codes an efflux pump that pumps the displaced macrolide out of the cell. Together, these genes belong to the two-gene efflux transport system of the ATP-Binding Cassette (ABC) superfamily and are responsible for type M resistance to macrolides (Iannelli et al., 2018). In *N. subflava,* the *mef(A)* is truncated and likely non-functional (de Block et al., 2021). This suggests that another efflux pump may be used by *N. subflava* to expel the dissociated macrolide. This function is may be carried out by the mtrCDE efflux pump. Interestingly, the *N. subflava’s* used in this study all contained the K823E *mtrD* mutant which is known to enhance the ability of the mtrCDE pump to export macrolides (Lyu et al., 2020).

Although it has been proven that interspecies recombination is successful between commensals and pathogenic *Neisseria in vitro*, we were unable to transform *msr(D)* into *N. gonorrhoeae* (Chen et al., 2020). There are a number of possible explanations for this finding. Firstly, the chromosomal organisation around *msr(D)* is very similar in the *N. subflava* donor and recipient strains but is divergent to *N. gonorrhoeae* strains (Supplementary Figures 3, 4). We have previously established that the core genome of the strains used in this study varies considerably between *N. subflava* and *N. gonorrhoeae* (De Block et al., 2021). This could affect efficient chromosomal integration of *msr(D)* and explain why interspecies transformation between *N. subflava* and *N. gonorrhoeae* was not successful (Qvarnstrom and Swedberg, 2006). Secondly, the relative frequency of the 12-bp DUS sequences varies considerably between *N. subflava* and *N. gonorrhoeae*. The 5’-ATGCCGTCTGAA-3’ DUS is more prevalent in *N. gonorrhoeae,* whereas the 5’-AGGCCGTCTGAA-3’ DUS is more prevalent in *N. subflava* (Supplementary Figure 4; Berry et al., 2013). These differences in the relative frequency of DUS-subtypes have been shown to influence the probability of transformation (Duffin and Seifert, 2010). This provided the rational for using dsDNA fragments containing *msr(D)* combined with the predominant *N. gonorrhoeae* DUS for the transformation experiments. However, this approach did not result in transformation. Thirdly, the differential DNA methylation pattern between species of *Neisseria* may result in the uptake of *msr(D)* containing DNA from *N. subflava* being toxic to *N. gonorrhoeae* but not *N. subflava* (Kim et al., 2020). Finally, the failure to transform *msr(D)* into *N. gonorrhoeae* may be due to limitations in our experimental approach. Although we used three different experimental approaches to transform *msr(D)* into *N. gonorrhoeae*, we did so in a limited number of strains. Furthermore, while we have previously been able to conduct successful transformation experiments with two of these strains of *N. gonorrhoeae* using the same experimental protocol, we did not include positive controls in the current experiments (Abdellati et al., 2019). These limitations mean that we cannot conclude that the *msr(D)* gene could not be transformed into *N. gonorrhoeae*. A further limitation of our study is the crude methods we used to measure the fitness cost associated with the acquisition of the *msr(D)* gene.

Another transformation pathway to evaluate in a future study is the transformability between different commensal strains. It may be possible to transform *msr(D)* from *N. subflava* to another commensal *Neisseria* species, such as *N. lactamica*, which is then able to transform the *msr(D)* in *N. gonorrhoeae* or *N. meningitidis*. For example, *N. lactamica* is known to be an efficient AMR donor to *N. meningitidis* (Chen et al., 2020).

Our study showed that intraspecies transformation of *msr(D)* under azithromycin pressure is very efficient within *N. subflava*. We were unable to transform *msr(D)* into *N. gonorrhoeae*. The limitations noted above mean that we cannot exclude the possibility of this occurring in the future.

## Data Availability Statement

The datasets presented in this study can be found in online repositories. The names of the repository/repositories and accession number(s) can be found at: https://www.ncbi.nlm.nih.gov/, PRJNA794044.

## Author Contributions

SA conducted the wet laboratory experiments. TB and NG conducted the bioinformatic analysis and wrote the first draft. CK, SA and SM-B conceptualized the study. SA, CD, JL, SM-B, IB, DB and CK reviewed and edited the manuscript. All authors contributed to the article and approved the submitted version.

## Funding

The study was funded by SOFI 2021 grant–‘‘PReventing the Emergence of untreatable STIs *via* radical Prevention, (PRESTIP).

## Conflict of Interest

The authors declare that the research was conducted in the absence of any commercial or financial relationships that could be construed as a potential conflict of interest.

## Publisher’s Note

All claims expressed in this article are solely those of the authors and do not necessarily represent those of their affiliated organizations, or those of the publisher, the editors and the reviewers. Any product that may be evaluated in this article, or claim that may be made by its manufacturer, is not guaranteed or endorsed by the publisher.
