# Supplementary material for: Successful Intra- but Not Inter-species Recombination of msr(D) in Neisseria subflava
Source: Front Microbiol. 2022 Mar 30;13:855482. doi: 10.3389/fmicb.2022.855482 (PMC9007320; doi:10.3389/fmicb.2022.855482)
Supplement: Supplementary file 1 [file Data_Sheet_1.PDF]

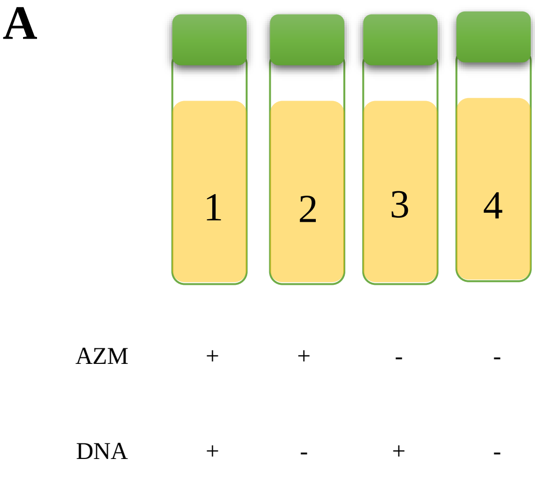

C

	1	2	3	4
AZM (mg/L)	1.5<br>3	1.5<br>3	-	-
DNA (150 ng/µl)	100 µl	-	100 µl	-
GCm	7.5 mL	7.5 mL	7.5 mL	7.5 mL

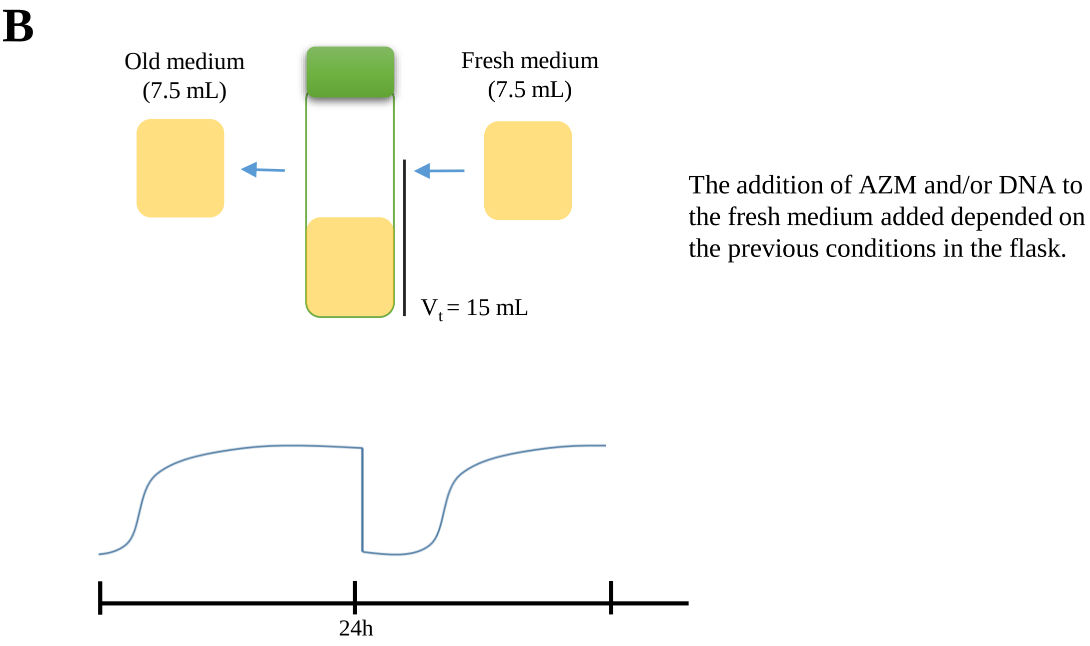

Supplementary figure 1. (A) Overview of the conditions (with or without DNA and/or azithromycin [AZM]) for inter-species transformation in morbidostat . (B) Diagram of the work process during the experiment – every 24 hours, 7.5ml medium was removed and replaced with fresh medium (V_t= Total Volume). (C) Concentrations/volumes of substances added daily per condition.
