# Supplementary material for: Successful Intra- but Not Inter-species Recombination of msr(D) in Neisseria subflava
Source: Front Microbiol. 2022 Mar 30;13:855482. doi: 10.3389/fmicb.2022.855482 (PMC9007320; doi:10.3389/fmicb.2022.855482)
Supplement: Supplementary file 2 [file Data_Sheet_2.PDF]

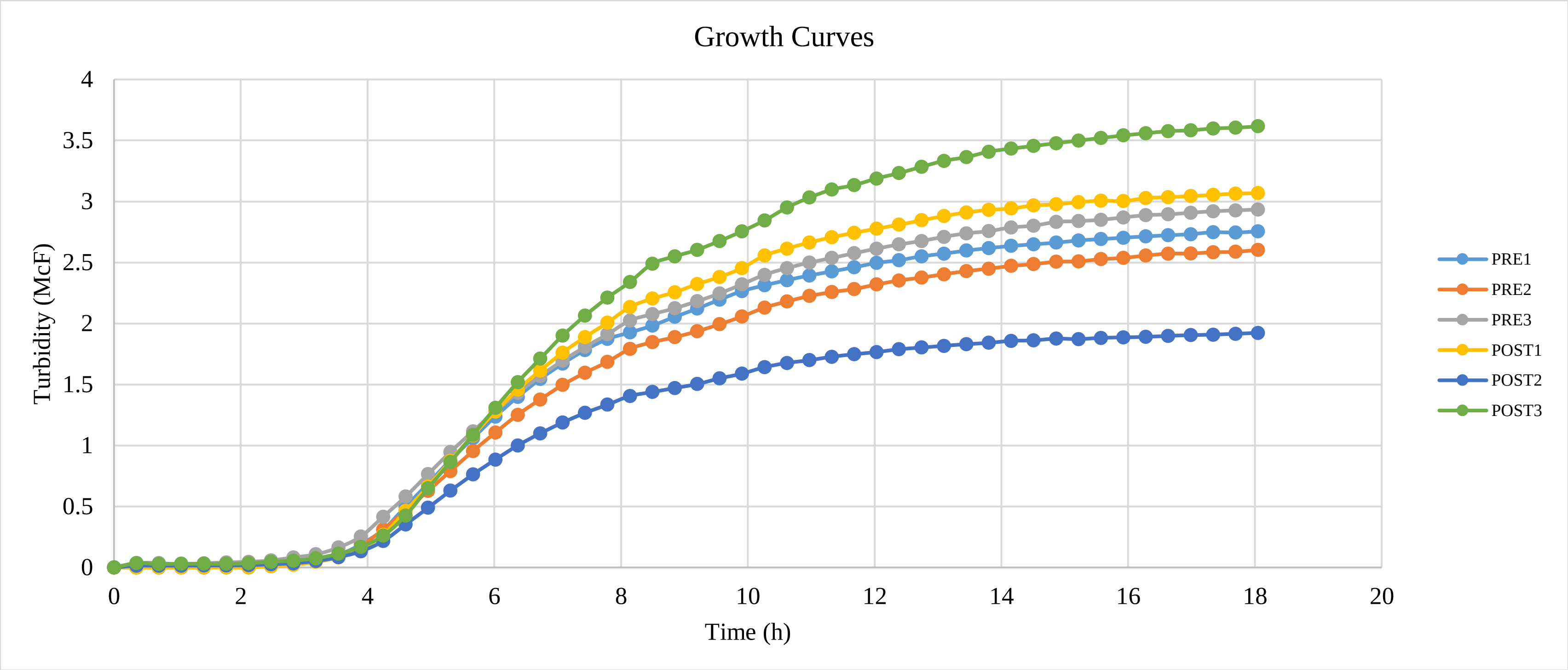

	GR1	GR2	GR3	Mean
**Pre-transformation**	0.6736	0.6326	0.6146	0.64
**Post-transformation**	0.6696	0.7145	0.6289	0.67

Supplementary figure 2. Growth curves and growth rates of the recipient and transformant strains for 18 hours. **(A)** The growth curves of the three recipients (PRE 1, 2 & 3) were compared to the same three transformants (POST 1, 2 & 3) for 18 hours. **(B)** The growth rates (GR) of each isolate, as well as the mean growth rate for the recipient and transformant strains. There was no significant difference in the growth rates between recipient and transformant (p-value = 0.3673; t-test).
