# Supplementary material for: Successful Intra- but Not Inter-species Recombination of msr(D) in Neisseria subflava
Source: Front Microbiol. 2022 Mar 30;13:855482. doi: 10.3389/fmicb.2022.855482 (PMC9007320; doi:10.3389/fmicb.2022.855482)
Supplement: Supplementary file 3 [file Data_Sheet_3.PDF]

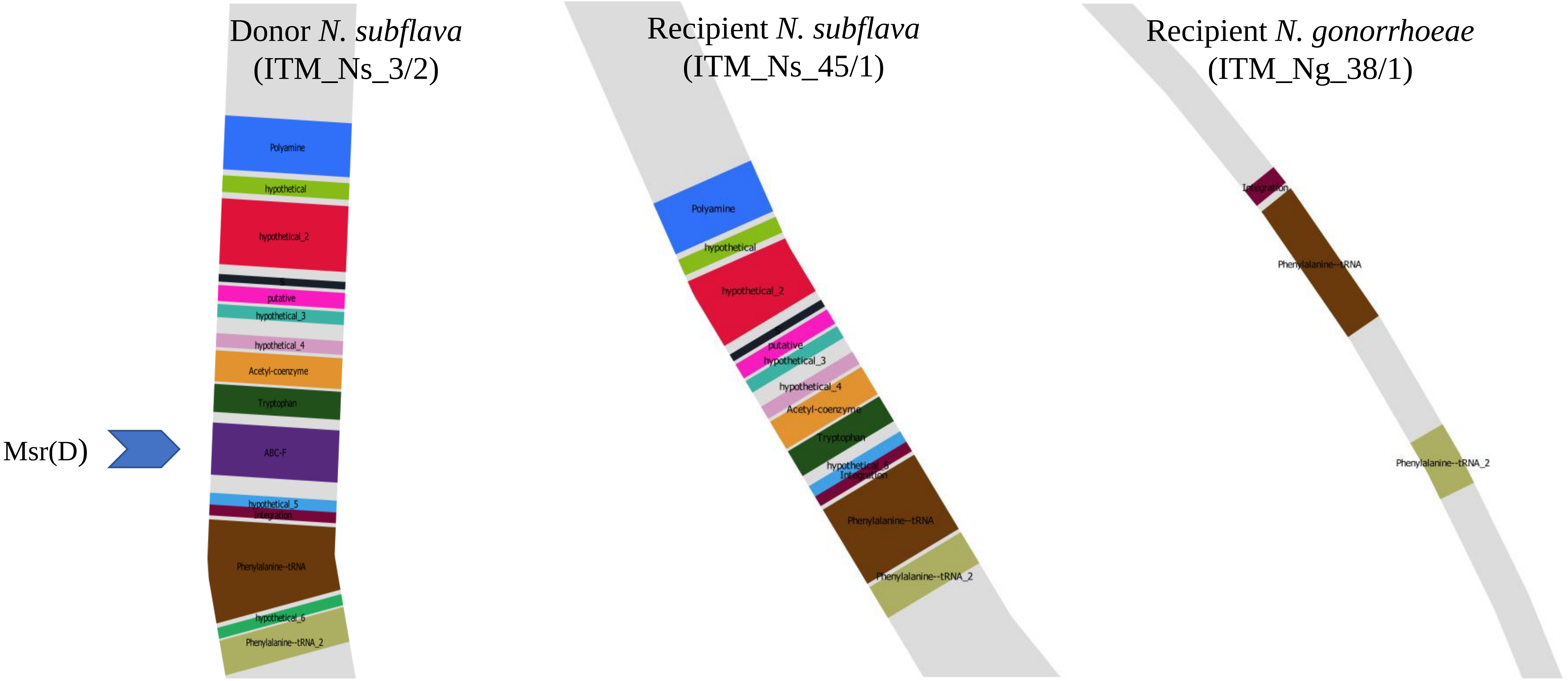

Supplementary Figure 3. Blast search result in Bandage based on genes around *msr(D)* showing the gene organization around *msr(D)* in donor *N. subflava*. Same gene list is used to visualize similar genes in recipients (same color correspond to same gene). Arrow indicates the *msr(D)* gene in donor strain.
