# Supplementary material for: Successful Intra- but Not Inter-species Recombination of msr(D) in Neisseria subflava
Source: Front Microbiol. 2022 Mar 30;13:855482. doi: 10.3389/fmicb.2022.855482 (PMC9007320; doi:10.3389/fmicb.2022.855482)
Supplement: Supplementary file 4 [file Data_Sheet_4.PDF]

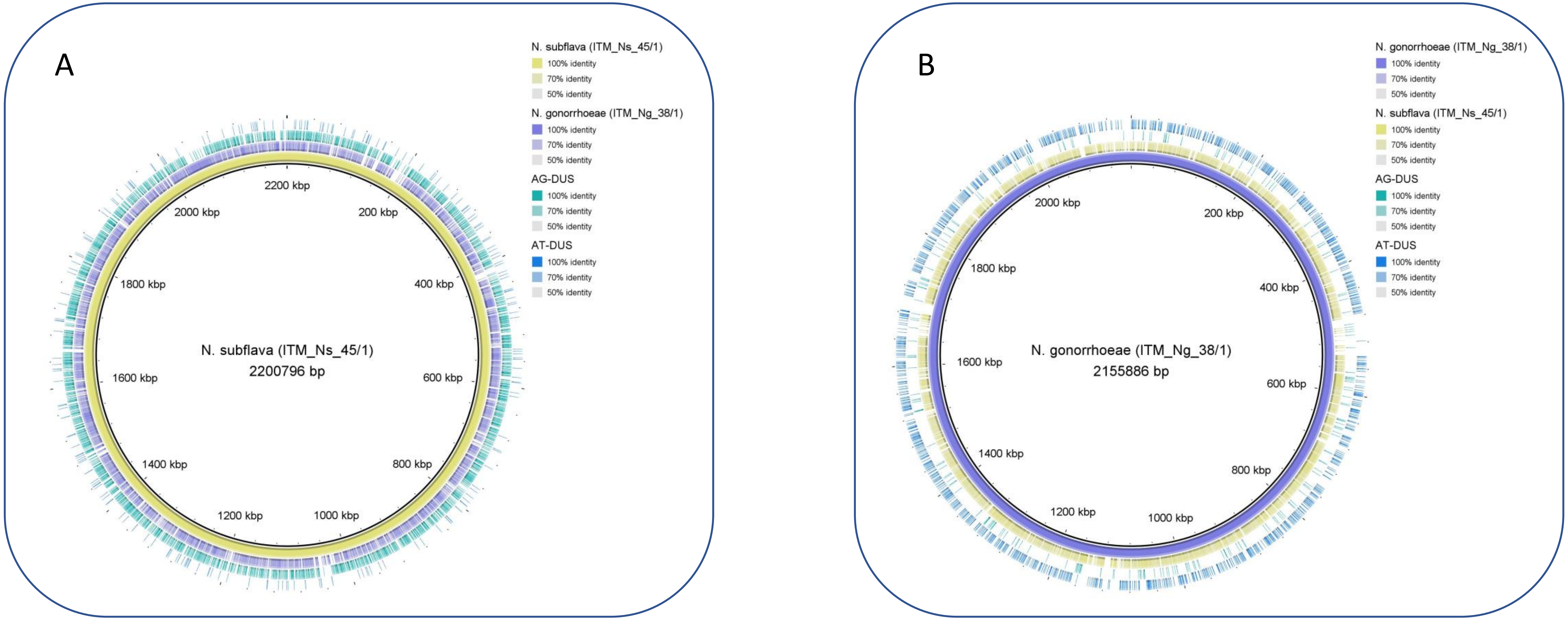

Supplementary Fig 4. Genome and DUS (AG-DUS: green circle; AT-DUS: blue circle) comparison of recipients (ITM_Ns_45/1 and ITM_Ng_38/1) used in this study visualazid in BRIG.

A. *N. subflava* (ITM_Ns_45/1) used as reference strain

B. *N. gonorrhoeae* (ITM_Ng_38/1) used as reference strain
